# Effect of Tempering Conditions on White Sorghum Milling, Flour, and Bread Properties

**DOI:** 10.3390/foods10081947

**Published:** 2021-08-21

**Authors:** Mohana Yoganandan, Scott R. Bean, Rebecca Miller-Regan, Hulya Dogan, Manoj Kumar Pulivarthi, Kaliramesh Siliveru

**Affiliations:** 1 Department of Grain Science & Industry, Kansas State University, Manhattan, KS 66506, USA; mohanay@ksu.edu (M.Y.); beckym@ksu.edu (R.M.-R.); dogan@ksu.edu (H.D.); pmanoj@ksu.edu (M.K.P.); 2 Grain Quality and Structure Research Unit, CGAHR, USDA-ARS, 1515 College Avenue, Manhattan, KS 66502, USA; scott.bean@usda.gov

**Keywords:** white sorghum, tempering, roller milling, kernel properties, flour properties, bread properties

## Abstract

The effects of room temperature water, hot water, and steam tempering methods were investigated on sorghum kernel physical properties, milling, flour, and bread-making properties. Overall tempering condition and tempering moisture content were found to have a significant effect on the physical properties. Milling properties were evaluated using a laboratory-scale roller milling flowsheet consisting of four break rolls and eight reduction rolls. Room temperature tempering (18% moisture for 24 h) led to better separation of bran and endosperm without negatively impacting flour quality characteristics i.e., particle size distribution, flour yield, protein, ash, damaged starch, and moisture content. Bread produced from the flour obtained from milling sorghum kernels tempered with room temperature water (18% m.c for 24 h) and hot water (16% m.c at 60 °C for 18 h) displayed better bread-making properties i.e., high firmness, resilience, volume index, higher number of cells, and thinner cell walls when compared to other tempering conditions. Room temperature water tempering treatment (18% m.c for 24 h) could be a better pretreatment process for milling white sorghum kernels without negatively impacting the flour and bread-making quality characteristics.

## 1. Introduction

Although sorghum has advantages from some nutrition aspects and a crop production standpoint, the commercialization of sorghum-baked products is limited due to the lack of standard milling processes. The technology of milling sorghum is not as developed as that of wheat, rice or maize [1,2]. Traditional methods of hand pounding and dehulling followed by hammer milling are still widely used for producing sorghum flour [3]. These methods result in coarser flour with low ash and oil content [4]. Moreover, abrasive decortication-hammer milling results in higher endosperm loss during bran removal [1]. The structural similarity of sorghum kernels with that of corn has enabled wet milling of sorghum using similar procedures to that of corn [5]. However, the smaller kernel size of sorghum when compared to corn does not support wet milling and has resulted in low extraction rate and loss of starch. In addition, the fragile and friable pericarp of sorghum may cause it to easily break during wet milling and produces undesirable specks of bran in the final product [6].

The similarity in kernel size of sorghum with that of wheat has led to the study of roller milling on sorghum [1]. Kebakile et al. [1] utilized two pairs of roller mills to mill sorghum using the first pair of rolls to break the sorghum kernels to smaller fragments with the bran intact and the second pair of rolls to grind the fragments to flour by separating it from bran particles. This research also compared the performance of roller milling sorghum with abrasive decortication-hammer milling where the former resulted in a higher production rate of fine-grained flour with greater oil, ash, and protein content than the latter. However, roller milled sorghum flour exhibited greater bran contamination when compared to sorghum flour produced by abrasive decortication-hammer milling.

In order to overcome bran contamination, the effect of tempering on sorghum kernels has been studied. Tempering, conditioning with water, is known to toughen the bran and soften the endosperm of grains to facilitate easy scratching of endosperm [7]. Additionally, tempering facilitates proper separation of bran and endosperm during milling. Tempering of sorghum positively influenced flour extraction rate and particle size distribution of flour during abrasive decortication-hammer milling [5]. The effect of different tempering conditions (room temperature water, hot water, and steam tempering) on physical and mechanical properties of red sorghum kernels and its milling quality were reported by Zhao and Ambrose [8,9]. In their study, the steam tempering was found to be efficient in strengthening the pericarp and softening of endosperm compared to the hot and room temperature water-tempering methods. Also, a study conducted by Chen et al. [10] to understand the effects of steam tempering on wheat quality found that the steam accelerated the migration of water through the kernel, resulting in a decreased tempering time period with improved rheological properties of the resultant wheat flour. 

Sorghum can be a good gluten-free replacement for those suffering from celiac disease, a disorder that induces gluten intolerance. Currently, the only cure for this autoimmune digestive disease is to consume a gluten-free diet throughout their life [11]. Hence, the increasing demand for gluten-free bread from the groups of celiac disease and the challenges involved in making gluten-free bread, have led to increased research on gluten-free bread production [12,13,14]. Much of the research on gluten-free bread production has focused on ingredients and product formulation, with less on the role of milling and flour properties. Flour particle size has been reported to play a significant role on gluten-free bread quality [15], which is dependent to a great extent on the milling technique performed. The other factors that affect the quality of bread are ash content of flour, which also depends on bran separation during milling, and water absorption capacity of flour during dough formation, which is affected by the damaged starch content in flour. These flour characteristics are affected by milling [16,17] to a great extent due to the involvement of size reduction and separation of bran and germ from endosperm to procure flour. Previous research has indicated that roller milling was more efficient in extracting sorghum flour and good baking properties [1,18].

Due to the interconnection between tempering, grain properties, milling, and flour properties, the goal of this project was to have a complete assessment of the impact of tempering on the roller milling, physical properties of grains, flour properties, and bread properties using a commercially available white sorghum grain. While some past research has also been conducted in this area, the current study provides additional information to the important area of milling, flour quality, and food production, and will serve to identify areas where improvements can be made to sorghum milling and flour quality.

## 2. Materials and Methods

### 2.1. Sorghum Sample Preparation

The white sorghum grains (45.4 kg) belonging to the species *Sorghum bicolor*, were procured from Nu Life Market, Scott City, KS, USA for this study. The grains were of food grade and are free from foreign matter such as dust, dirt, stones, and chaff with an initial moisture content of 13.46% wet basis (w.b.). The initial moisture content (m.c) of sample was measured using ASABE Standard S352.2 method [19].

### 2.2. Tempering

#### 2.2.1. Room Temperature Tempering

Samples were tempered with a predetermined amount of distilled water (water available at room temperature) [20]. The tempering process was carried out in a rotating drum to bring them to final m.c of 16% and 18% (w.b.). These moisture contents were chosen from previous research conducted by Zhao and Ambrose [8] and Abdelrahman and Farrel [21] based on higher bran separation of sorghum. The room temperature water tempered samples were tightly air packed in zip lock bags and held at room temperature for 24 h for uniform distribution of moisture throughout the samples.

#### 2.2.2. Hot Water Tempering

The samples were treated with the pre-calculated amount of room temperature distilled water in the rotating tempering drums to bring them to a final m.c of 16 and 18% (w.b.). The treated samples were then placed into glass beakers and sealed with aluminum foil to prevent water evaporation. The beakers were next placed in a hot water bath at 60 °C for 12, 18, and 24 h [22]. The glass beakers were shaken intermittently for every 30 min. After conditioning the samples for the respective time periods, the samples were allowed to cool at room temperature prior to the evaluation of the kernel properties.

#### 2.2.3. Steam Tempering

The samples were tempered with steam at 20 psi for 5, 10, and 15 s in a hollow container with a screw passing horizontally through the inside of the container. The horizontal screw was used to mix the sorghum samples as steam passed through the inlet, which allowed the uniform distribution of steam throughout the sample. After the samples were steam treated, the samples were spread on flat trays for about 8 h to evenly dry the samples. The samples were then transferred to zip lock bags and stored at 5 °C until needed.

### 2.3. Kernel Properties Measurements

#### 2.3.1. Density Measurements

The bulk density of the tempered and untempered white sorghum samples was measured using Winchester cup arrangement (Seedburo Equipment Co., Des Plaines, IL, USA). The samples were allowed to fall freely into a cup of volume 1 pint (1 pint = 4.732 × 10^−4^ m^3^) from a funnel set at a height of 10 cm from the mouth of the cup. The cup filled until excess sample began to overflow, and excess grain was removed by passing a scrapper in a zigzag motion over the cup. The bulk density of the samples was then calculated from the weight of the sample and volume of samples in the cup of known volume. Tapped density of both tempered and untempered sorghum samples was measured using Autotap Density Analyzer (Quantachrome Instruments, Boynton Beach, FL, USA). A total of 100 ± 1 g of grain sample was filled into the cylinder of known volume, and the cylinder was then tapped using Autotap Density Analyzer for 750 times. The tapped density was calculated as the ratio of the mass of sample taken to the final volume after tapping.

A gas pycnometer (AccuPync II 1340, Micromeritics, Norcross, GA, USA) was used to measure the true density of both tempered and untempered white sorghum samples. Helium gas was diffused into the chamber to fill the entire volume of the chamber. The volume occupied by the sample and the helium gas in the closed chamber was measured by the pycnometer. The true density of the sample was then determined as the ratio of the mass of the sample taken to the volume of sample occupied by the solid particles in the chamber.

#### 2.3.2. Grain Physical Traits

Physical properties (mean kernel hardness, mean kernel weight, and mean kernel diameter) of untreated and tempered grain were measured by both the single kernel characterization system (SKCS) and abrasive hardness index (AHI) using a tangential abrasive dehulling device (TADD) [23].

#### 2.3.3. Angle of Repose

For the angle of response measurements, 450 g of tempered and untempered white sorghum sample was allowed to freely fall onto a flat surface from a funnel held at a height of 10 cm above the horizontal flat surface. The diameter (*d*) and height (*h*) of the formed grain pile was measured and the angle of repose (*ϴ*) was calculated using Equation (1).

(1) $$ϴ=\tan^{-1}\left(\frac{2h}{d}\right)$$

#### 2.3.4. Coefficient of Static Friction

The coefficient of static friction was measured for tempered and untempered white sorghum samples against galvanized steel as described in Subramanian and Viswanathan [24] and Patwa et al. [25].

#### 2.3.5. Coefficient of Rolling Friction

Approximately 150 g of sample was placed in a two-sided open cylinder resting on a horizontal galvanized steel plate fitted on a wooden board. The cylinder was removed to allow the sample to form a stationary cone on the plate. A magnetic compass was placed on the horizontal plate and calibrated to 0⁰ or no inclination. The plate attached to the board was tilted on one side by rotating the screw on the opposite side of the board. The angle (*α*) at which the sample begins to slide down the plate was measured to calculate the coefficient of rolling friction (*r*) using Equation (2).

(2) $$\mu_{r}=\tan\left(\alpha \right)$$

### 2.4. Roller Milling Procedure

**Figure 1:**
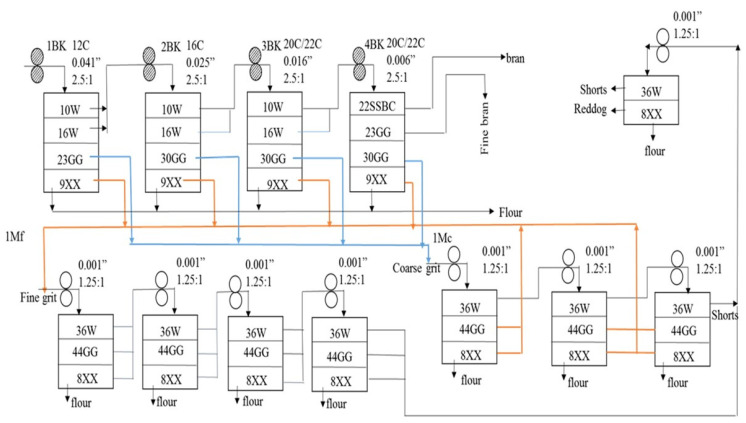
Sorghum Roller Milling Flowsheet (BK: Break, C: Corrugation, Mf: Fine grit milling, Mc: Coarse Grit Milling).

Preliminary milling trials were performed based on the understanding of milling basics and following a trial and error approach using tempered white sorghum kernels. The developed flow sheet (Figure 1) for roller milling of sorghum consisted of four pairs of break rolls and eight pairs of reduction rolls along with sieving. The first two pairs of break rolls broke the kernel to smaller particles keeping the bran intact, and the last two break rolls scraped the bran from the endosperm. Based on the particle size, the grits were classified into fine and coarse. The classified grits were reduced to flour using respective reduction rolls. Among the reduction rolls, three pairs of reduction rolls were used for milling coarse grits and five pairs were used for milling fine grits. The milling outcomes from each pair of rolls was passed through a set of sifters (Figure 1) and the milling stocks from each sieve were collected and weighed to separate the outcomes based on their size.

The break rolls had a speed differential of 2.5:1 and roll disposition of dull-to-dull, whereas speed differential of the reduction rolls was maintained at 1.25:1. The roll gaps for break and reduction rolls were determined based on preliminary trials (Figure 1). Based on the preliminary trials, the selection criteria for flow sheet were narrowed down to achieve maximum flour extraction yield and lower ash content. The milling process was performed in triplicates for every tempering treatment. The fractions collected from the milling process were bran, fine bran, shorts, red dog, and flour. The milling fractions were collected as shown in Figure 1. The yield (%) of each milling fraction was calculated using Equation (3).

(3) $$\mathrm{Yield}\;\left(\%\right)=\frac{\mathrm{Weight}\;\mathrm{of}\;\mathrm{total}\;\mathrm{milling}\;\mathrm{fraction}\;\mathrm{obtained}\;\mathrm{from}\;\mathrm{milling}\;\left(g\right)}{\mathrm{Initial}\;\mathrm{weight}\;\mathrm{of}\;\mathrm{grain}\;\mathrm{taken}\;\left(g\right)}\times 100$$

The sieve sizes used for separating the milling outcomes are explained in Table 1.

### 2.5. Flour Properties

#### 2.5.1. Flour Particle Size Distribution

The particle size distribution of white sorghum flour samples was determined by Ro-Tap analysis according to ASABE Standard S319.4 [26]. The weight of all the empty sieves was recorded and flour (100 ± 0.1 g) was added to the topmost sieve and sieved for 10 min. The mass of sample retained on each sieve was recorded and the particle size distribution curve was plotted for both the samples.

#### 2.5.2. Flour Chemical Composition and Physical Properties

The proximate composition of the sorghum flours was determined following the standard procedures AACC 76.33 [27] using SDmatic^®^ (Chopin Technologies, Villeneuve-la-Garenne, France), which was followed to estimate the damaged starch in flour. Total starch (%) of flour samples was analyzed using Megazyme Total Starch Assay Procedure (Megazyme International Ireland Ltd., Bray, Ireland) (AACCI Method 76-13.01). Protein (%), crude fat (%), crude fiber (%), and ash (%) content were analyzed using AACC Method 46-30.01, AACC Method 30-25.01, AACC Method 32-10.01, and AACC Method 08-01.01, respectively [27].

The moisture content of the flour was measured in wet basis using standard method, AACC 44-15.02 [27]. Bulk, tapped, and true density of the milled white sorghum flour was measured according to the same techniques as for grain mentioned previously. The color of white and waxy white sorghum flour was measured using HunterLab MiniScan EZ 45/0° Spectrophotometer (Hunter Associates Laboratory Inc., Reston, VA, USA).

### 2.6. Bread Formulation and Production

#### 2.6.1. Bread Formula

Three loaves of bread were made from each tempering treatment. The following formulation was used to make the sorghum bread with all ingredients in flour weight basis (fwb) (%): milled sorghum (white and waxy white sorghum) flour (90%), white rice flour (Bob’s Red Mill Natural Foods, Inc., Milwaukee, OR, USA) (10%), refined white sugar (6%), emulsified shortening (5%) (Sweetex, Stratas Foods, Memphis, TN, USA), xanthan gum (3%) (Judee’s Gluten Free, Columbus, OH, USA), double acting baking powder (Monocalcium phosphate and sodium aluminium sulfate) (8%), common salt (1.5%), and distilled water (110%) [28].

The batter was made in KitchenAid 6 Quart Professional 600 mixer with a coated flat beater (Whirlpool Cooperation, Benton Charter Township, MI, USA). The shortening and dry ingredients were weighed and first added to the mixing bowl after which the required amount of distilled water was added to the same. All ingredients were mixed with the flat beater paddle on speed 1 for 30 s. The batter was scraped down with a rubber spatula and mixed on speed 2 for 1.5 min. The final batter was weighed and transferred into greased metal pup loaf baking pans (15 × 9 × 5 cm^3^) and baked in a rotary baking oven (Reed Oven Co., Kansas City, MO, USA) at 204 °C for 30 min. Loaves were withdrawn immediately from the pans after removal from the oven and cooled for 2 h on wire racks to bring to room temperature. After cooling the breads, they were weighed to evaluate the loaf weight and bagged individually in polyethylene bags. The packed loaves were stored at room temperature overnight.

#### 2.6.2. Bread Characterization

Volume index, internal crumb analysis, and crumb texture were analyzed 24 h after baking. The bake loss was calculated right after the loaves cooled down to room temperature and before packing them into polyethylene bags. The batter weight was evaluated for the batter from each tempering treatment, and the bread loaves made from the same treatment were weighed after removing them from the oven and cooling to room temperature. The bake loss for each loaf was evaluated using Equation (4).

(4) Bake loss (g)=(Batter weight)(g)−(Loaf weight)(g)

Volume index was determined using a cake template as described in AACC International Approved Method 10-91.01 [27] using a 3-cm-wide slice cut from the long dimension from the center of each loaf [28]. The internal crumb structure of both white and waxy white sorghum bread was evaluated using C-Cell (Calibre Control International Ltd., Appleton, Warrington, UK) according to AACC Method 10-18.01 [27]. Image analysis software (C-Cell Software Version 2.0) was used to quantify the crumb cell characteristics. Three square slices were analyzed for each treatment. Resilience and firmness of the sorghum breads were evaluated using a TA.XT *Plus* Texture Analyzer (Texture Technologies Corp., Scarsdale, NY/Stable Micro Systems, Godalming, Surrey, UK) in accordance with AACCI Method 74-10.02 [27].

### 2.7. Experimental Plan and Statistical Analysis

The study was a completely randomized design with 11 treatments as follows: room temperature water (final m.c 16% and 18% w.b.); hot water (final m.c 16% and 18% w.b. for 12, 18, and 24 h for each final m.c); and steam for 5, 10, and 15 s. The results were analyzed for statistical significance using the PROC GLM (general linear models) procedure in SAS (Statistical Analysis System) (ver. 9.3, SAS Institute, Inc., Cary, NC, USA). Based on ANOVA, significant differences (α = 0.05) among the treatments were reported as significant at *p* ≤ 0.05.

## 3. Results and Discussion

To evaluate how tempering may impact the physical properties of sorghum and subsequent milling quality and flour and bread quality, a commercial sample of white sorghum grain was tempered using different conditions and results were evaluated. Sorghum is known to be a diverse crop that can have a wide range of physical and chemical properties, which could impact tempering, milling, and flour quality. Compared to other crops used for flour production, there has been relatively little research conducted to standardize sorghum flour milling procedures. Thus, from a practical standpoint, a commercially available white sorghum sample was selected for this research to identify tempering methods that can be used in future research with additional samples.

### 3.1. Effect of Tempering on Physical Grain Properties

The bulk density for white sorghum kernels decreased when tempered (Table 2). This was attributed to penetration of water into the kernels, which increased the weight of the kernels at a slower rate when compared to the volumetric expansion of the kernel [29]. There was no significant difference in between the bulk density values of room temperature water and hot water tempered sorghum kernels, whereas the bulk density values of steam tempered sorghum kernels were much lower than the other two treatments (Table 2). This could be due to the increase in moisture content of sorghum kernels during the steam tempering.

Tapped density decreased for both room temperature water and hot water tempering as tempering moisture content increased (Table 2). Likewise, the duration of steam tempering significantly decreased the tapped density of the kernels (Table 2). Again, this may have been due to the increased moisture content of the steam-tempered samples as discussed with respect to bulk density. Patwa et al. [25] reported a similar negative relationship between moisture content and tapped density for wheat kernels. The increase in the moisture content during steam tempering could have reduced the flowability of the kernels, which in turn left void spaces between kernels even after tapping, consequently reducing the tapped density of the kernels.

Steam-treated sorghum exhibited the lowest true density values respectively when compared to kernels tempered with room temperature and hot water (Table 2). The sorghum sample showed a decreasing trend in true density with increasing moisture content when tempered with room temperature and hot water. A subsequent increase in moisture content of the grain was observed upon increase in the steam tempering time from 5to 15 s. Consequently, the true density decreased from 1351.07 kg/m^3^ to 1325.13 kg/m^3^. The results obtained from this density study agreed with results obtained for coriander seeds [30] and soybean [31].

The dehulling characteristics of grains can be predicted using the abrasive hardness index, AHI [9,23]. There were no significant differences in the AHI values between untempered and tempered kernels (Table 2). This implies that tempering method, moisture content, and tempering time had a negligible influence on the force required to abrade the outer surface of sorghum kernels. Reichert and Ehiwe [32] reported that a seed’s resistance to split (or its hardness index) and binding strength of the seed coat/pericarp to the seed cotyledon/endosperm adversely affected the dehulling capacity of the seed. The results obtained from this study showed that the adherence between the pericarp and endosperm was strong even after tempering.

Tempering conditions had various effects on grain SKCS hardness index values. There was no significant difference in the HI values upon cold water tempering. Tempering with room temperature water had a significant effect on SKCS-HI values (Table 2). Likewise, tempering with hot water at 16% m.c also did not significantly impact grain hardness. However, tempering with hot water at 18% m.c resulted in a slightly significant decrease in SKCS-HI (Table 2). Steam tempering also decreased the hardness index of sorghum kernels. With an increase in tempering time, the moisture content of the kernels increased and the hardness index decreased. Similar trends, i.e., the negative correlation between the moisture content and grain hardness have been reported for sorghum [9]. This agrees with the AHI measurements discussed above, where abrasive hardness index was found to decrease during steam tempering.

Mean kernel diameter and mean kernel weight of steam-tempered white sorghum were greater than untempered kernels (Table 2), again likely due to water penetration into the intracellular spaces of the kernel during tempering [29]. It is evident that steam-tempered white sorghum kernels had a relatively greater mean kernel weight and diameter when compared to all the other tempering conditions, again due to greater water penetration into the kernels during steam tempering than other methods (Table 2).

In addition to density measurements and grain hardness and physical size measurements, tempering also influenced frictional properties of the grain. Duration of tempering or heat treatment did not significantly influence the coefficient of static friction (Table 3). The greater amount of water present in the kernels tempered with steam possibly increased the adhesive forces between the kernel and the surface of galvanized steel, causing it to have a high coefficient of static friction [23,30]. However, the increase in static coefficient of friction of the kernels may not be solely due to increasing moisture content as surface characteristics of the kernel could have also possibly played a role in the increase.

Steam-tempered white sorghum displayed the highest rolling coefficient of friction when compared to other treatments. The rolling coefficient of friction increased with increment in the moisture content when treated with room temperature or hot water (Table 3). These results agreed with the results obtained from coefficient of rolling friction studies on coriander seeds [29], fenugreek [33], and millet [34]. The increase in rolling coefficient of friction may have been due to an increase in cohesive forces developed between the kernel and surface with the addition of water during tempering, which made the grains rougher than usual and reduced their sliding characteristics [29].

No significant trend was observed in the rolling coefficient of friction with increasing heat treatment time. However, a distinct increase was observed in this frictional property when white sorghum was steam tempered at 20 psi from 5 to 15 s. The hollow cylinder containing the grains was removed for the sample to form a cone on the flat galvanized steel plate before the plate was inclined to calculate the rolling coefficient of friction. From the angle of repose results (Table 3), steam-tempered (for 15 s) kernels displayed the greatest angle of repose, which can be attributed to the reduced flowability between the kernels itself causing greater force required to overcome the flowability between the kernels and overcome the cohesive forces between the kernels and the surface. This could be attributed to the fact that white sorghum tempered with steam at 20 psi for 15 s exhibited the highest rolling coefficient of friction (0.32) when compared to all other treatments. The structural changes caused by steam tempering at the mentioned pressure and duration could also have possibly caused the high rolling coefficient of friction by making the surface rougher and offering greater resistance.

### 3.2. Effect of Tempering on Flour Properties

The impact of the various tempering treatments on sorghum flour milling as conducted in this research is shown in Table 4. Steam tempering for 10 s and 15 s produced the least amount of flour when compared to other tempering treatments. There was no significant (*p* < 0.05) difference in flour yield with increasing tempering moisture content or type of tempering. Bran extraction from the grain during milling increased with increasing moisture content when tempered with room temperature, hot water or steam (Table 4). Steam tempering for 10 s and 15 s provided the most efficient separation of bran using the milling process described in this paper. Though fine bran was an insignificant quantity of the milling fractions, it can potentially contaminate flour. Steam tempered (15 s) of the white sorghum sample used produced maximum extraction of fine bran when compared to other treatments (Table 4). Shorts are a by-product of milling coarse and fine grits, which are in the form of flaked endosperm containing some bran, flour, and germ [33]. A negative relationship was observed between the rate of production of shorts and tempering moisture content when tempered with room temperature water (Table 4). The red dog fraction is also a by-product of milling grain, which consists of bran, germ, and flour. The sorghum sample used in this research displayed a decrease in the production of red dog with an increase in tempering moisture content while using room temperature water (Table 4). However, no specific trend was observed with the yield of red dog upon increasing the tempering time and moisture content of hot water tempering. The production of red dog reduced gradually when steam tempering time increased from 5 s to 15 s. Steam tempering to 15 s exhibited the lowest value of 10.05% red dog yield from the white sorghum tested.

**Figure 2:**
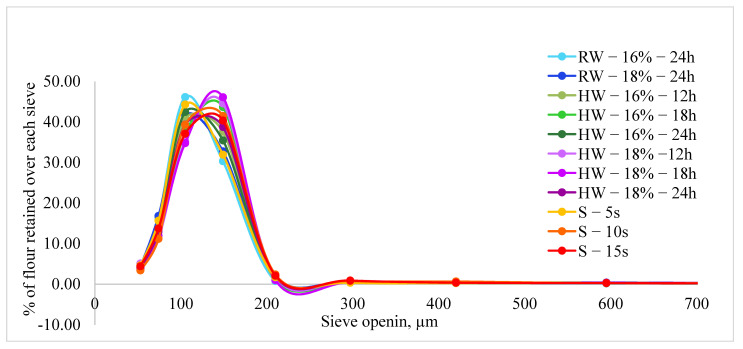
Particle size distribution of the white sorghum flour.

The particle size distribution of the flour is dependent on the milling characteristics, tempering conditions, and kernel properties. From the particle size analysis, it was observed that more than 98% of the particles were below 212 µm (Figure 2) and thus identified as “flour” in this project as defined per the Code of Federal Regulations [35]. From Figure 2, it is evident that the percentage of flour below 105 µm decreased with increasing tempering moisture content when treated with room temperature water. In the case of hot water tempering, varying the tempering time or moisture content did not make any observable trend in the particle size distribution (Figure 2). Steam tempering to 10 s and 15 s produced lower levels of flour ≤ 105 μm than other treatments. Changing the tempering condition from room temperature water to steam reduced the production of fine-grained flour (≤105 μm).

### 3.3. Effect of Tempering on Proximate Composition of White Sorghum Flour

Room temperature, hot water, and steam tempering had a significant impact on the total starch content (Table 5). The flour obtained from steam-tempered treatment resulted in higher total starch values (86.69 to 88.20 g/100 g) compared to the room temperature tempering (81.38 to 82.91 g/100 g). However, Zhao and Ambrose [8] reported a negligible effect on total starch content with varying time and heat treatments for red sorghum flour. Tempering moisture content or time did not affect the protein content of white sorghum flour produced (Table 5). Similar results were reported by Bai et al. [34] for chickpea and barley flour. However, steam tempering for 10 and 15 s reduced protein content of the sorghum flour. The protein content of sorghum kernel is greater in the corneous endosperm than the floury endosperm [36]. The lower protein content of white sorghum flour produced from steam-tempered kernels suggests that these tempering conditions caused portions of the corneous endosperm and outer layers of hard endosperm to be lost (separated along with the bran) during milling, i.e., steam tempering impacted the breakage pattern of the kernels, thus leading to higher levels of bran composition.

Ash content determination in any particular flour is comparatively an accurate indicator of the separation of endosperm from pericarp and germ [37]. The white sorghum grain used in this research contained 1.67% ash content. When the sorghum sample was treated with room temperature water for 24 h, the flour produced had the lowest ash content (0.63%). Steam tempering was also efficient in producing flour with less ash (Table 5). The heat and moisture combination during steam tempering could have toughened the bran and softened the endosperm, resulting in lower accumulation of bran components in flour, and thereby resulting in lower ash content. The ash content of the milled sorghum flour from all tempering treatments was significantly higher than reported by Zhao and Ambrose [8] using roller milling of a red sorghum (0.33% to 0.43%), although it was lower than reported by Liu et al. [38] (1.25% to 1.41%) using abrasive decortication-hammer milling on white and red sorghum. This suggests that roller milling is a better milling technique to reduce ash content in flour or possible differences in grain composition and structure between the studies.

The major insoluble fiber component of sorghum is cellulose, which varies from 1.19 to 5.23% in sorghum and has been reported to be reduced during milling [39]. As expected, the results of the current study also found a reduction in crude fiber of flour compared to whole grains (Table 6). White sorghum flour displayed no increasing or decreasing pattern in crude fiber content with tempering moisture content or time when tempered with room temperature water, hot water, or steam.

Hot water tempering did not reveal any specific trends with regards to fat content in the flour fraction (Table 5), similar to results found previously [8]. However, sorghum flour showed a decrease in crude fat content with increasing moisture content during room temperature and steam tempering. This could be associated with increasing bran yield during milling of white sorghum tempered at the mentioned conditions. As lipids in sorghum are located in the scutellum of the germ and aleurone layer of the kernel [40,41], these components will be removed during milling and located in the bran fraction. Lipids in sorghum flour were reported to decrease significantly due to bran removal [1] and its composition was reported to be changed during milling and size reduction [39].

Damaged starch in milled flour increases the water-holding capacity of flour by increasing the water absorption. Higher concentration of damaged starch also increases the starch digestibility during the dough making due to higher levels of starch exposed for hydration and enzymatic action [42]. Room temperature water tempering produced sorghum flour with the lowest damaged starch. Damaged starch levels in the sorghum flour did not show any significant trend with increasing tempering moisture content or time period when treated with room temperature and hot water (Table 5).

Bulk density of flour plays an important role in developing packaging material. Lower density flours occupy greater space, consequently increasing packaging material per unit weight. Tapped density is expressed as compacted bulk density. True density is the density of the material with respect to the actual volume occupied by it excluding closed and open pores. The bulk, tapped, and true density of the white sorghum flour produced in this research increased with increasing tempering moisture content when tempered with room temperature and hot water (Table 7). Similar results were found by Subramanian and Viswanathan [24] on millet flours and Bengal gram flour [43]. Tempering time did not show any effect on the densities of flour except when tempered with steam (Table 7). The increase in the densities with tempering moisture content was attributed to the increasing cohesive forces between flour particles with increasing moisture content. Bulk density of flour can also be affected by flour particle shape, which could cause the flour particles to compact together. The inclusion of water during tempering caused the endosperm particles in the flour to expand, in which its mass expansion was greater than its volume expansion, thereby increasing the density of flour [25]. Steam tempering white sorghum to 15 s produced flour with the highest bulk (484.06 kg/m^3^), tapped (581.40 kg/m^3^), and true (1478.83 kg/m^3^) density when compared to the other treatments.

The lightness value of flour can be related to the bran contamination in flour. Along with the ash content, Kim and Flores [44] used color and bran speck counts to determine the bran contamination in wheat flours. Thus, flour color was analyzed in this project to determine how color related to fractionation during milling. Flour showed increases in lightness values when tempering condition changed from room temperature water to steam (Table 7). Steam-tempered (for 10 s and 15 s) white sorghum produced flour with the greatest lightness value (or brightest color) due to efficient separation of bran from endosperm when compared to other tempering conditions. This is also supported from the final bran quantity obtained by milling steam-tempered (10 s and 15 s) white sorghum using the developed flowsheet (Figure 1). Tempering time and tempering moisture content did not seem to influence the lightness value of the obtained flour. 

Analysis of flour color indicated a slant towards the red side due to all positive a-values (Table 7). The redness of the flour decreased with increasing moisture content when tempered with room temperature water. However, no such trend was observed in hot water-tempered white sorghum. Flour produced from steam-tempered white sorghum for 15 s displayed the lowest a-value. This result also aligned with the greatest lightness value of the flour obtained from the same treatment.

The positive b-values indicated that the flour obtained from all tempering treatments fell in the yellow range (Table 7). The yellow color of flour could be attributed to the presence of carotenoids in the proteinaceous matrix of endosperm [45]. This explained the lowest b-value in sorghum flour obtained from steam-tempered white sorghum for 10 s and 15 s due to the maximum amount of bran (with vitreous endosperm) being removed from the grain when tempered with the above two treatments. Cold and hot water tempering moisture content and time did not provide any significant trend in the b-value of white sorghum flour.

### 3.4. Effect of Tempering on Sorghum Flour Bread Properties

The type of tempering, increasing moisture content, or tempering time, did not affect bake loss for white sorghum breads (Table 8). Steam-tempered and hot water-tempered white sorghum displayed bread with the greatest (178.86 mm) and lowest volume index (163.92 mm) respectively. Tempering moisture content and tempering time displayed no effect on the volume index of bread (Table 8). However, flour procured from white sorghum grain tempered with room temperature water for 24 h (18%) produced bread with the highest volume index. Bread volume is an important quality attribute of bread and depends on several factors like viscosity of the batter and the presence of surface-active components [46], thus room temperature tempering may be best in situations where sorghum flour will be used for gluten-free bread production.

The slice brightness of white sorghum bread decreased from 128.84 to 123.5 to 113.98 when the tempering treatment changed from steam to room temperature tempering to hot water, respectively. Steam-tempered white sorghum produced flour with the lowest bran contamination and ash content (Table 4 and Table 5), resulting in the bread from these flours having the brightest color. A strong negative correlation between slice brightness and ash content of flour (*r* = −0.84) indicated that the slice brightness of the bread crumb was significantly influenced by the ash content in the flour. Thus, white sorghum tempered with steam for 15 s produced flour with lowest bran contamination and highest lightness value. However, tempering time and tempering moisture content did not affect the slice brightness of the sorghum bread (Table 8).

There was no observable relationship between type of tempering and the average number of crumb cells in the sorghum bread (Table 8). Similar to other crumb grain characteristics, change of tempering conditions from cold water to hot water or steam, tempering moisture content, and tempering time did not display any effect on the cell diameter and cell wall thickness of the baked white sorghum bread (Table 8).

The firmness of white sorghum bread decreased with the tempering condition changing from room temperature water (1359.83 g) to hot water (1229.30 g) to steam (1015.92 g). Crumb firmness of gluten-free bread was reported to increase with increasing amount of protein in the flour [47]. Flour from steam-tempered white sorghum had the least amount of protein when compared to the flour produced from other tempering treatments (Table 5) and could account for the low firmness of sorghum bread produced from the steam-tempered sorghum treatment. A strong positive correlation was determined between the protein content of the flour and firmness of the bread (*r* = 0.79). No relationship was observed between slice resilience and type of tempering, tempering time, or tempering moisture content (Table 8). 

## 4. Conclusions

The study revealed that steam tempering has the greatest impact on grains physical properties of all the different tempering conditions tested. Steam tempering of sorghum grain at 20 psi for 15 s produced the desired physical kernel properties such as lower bulk density; higher angle of repose and coefficients of friction; and lower hardness index for efficient handling, transportation, and processing, however, the high moisture content resulting from this treatment is unsafe and risky for storage. Though the steam-tempered kernels required the least time to temper, proper drying techniques and storage conditions would be essential to maintain the quality of the grain.

The developed flowsheet using laboratory-scale roller milling produced an average of 60.24% flour from the white sorghum used to evaluate the tempering treatments. Steam tempering for 15 s produced white sorghum flour with greater total starch, least bran contamination, brightest color, and low ash content. However, this tempering method produced the lowest flour yield, protein content, and high damaged starch. Tempering white sorghum with room temperature water for 24 h to a final moisture content of 18% (w.b.) produced better flour yield without compromising the protein content of flour and with the lowest ash content and damaged starch. More than 98% of the total milled sorghum flour produced from all tempering methods was less than 212 μm and as such could be claimed to be flour under the current CFR. Tempering white sorghum with room temperature water for 24 h could be a suitable tempering method to obtain good flour yield and flour characteristics. However, the scaling up, cost estimation, and energy consumption assessment of the developed technique need to be evaluated.

Tempering was also found to influence flour characteristics and, subsequently, sorghum bread characteristics. For example, sorghum bread made from flour using steam-tempered grains produced bread with higher brightness, which was attributed the lowest bran and ash content. Both room temperature-tempered grain (18% moisture) and grain tempered with hot water for 18 h (16% moisture) produced bread with a greater number of crumb cells and high firmness and resilience. As tempering conditions influenced flour properties such as starch damage, color, etc., tempering may be one avenue to impact sorghum food product quality, especially when considering interactions with ingredients such as hydrocolloids. Manipulating starch damage to alter water absorption and batter viscosity for example, may help reduce the use of expensive ingredients such as hydrocolloids or other additives in gluten-free breads. Further research on the interactions between tempering, milling, and flour functionality would benefit the gluten-free flour-baking industry.

## Figures and Tables

**Table 1:** Sieve sizes used in Figure 1 for separating milling outcomes.

Sieve Used	Sieve Opening (µm)
10W	2030
16W	1180
22SSBC	977
23GG	900
30GG	600
36W	478
44GG	425
8XX	193
9XX	150

(W: wire; GG: grits gauze; SSBC: stainless steel bolting cloth; XX: silk cloth).

**Table 2:** Moisture content, densities (bulk, tapped and true density), and single kernel characterization system properties of sorghum.

Tempering Condition	Moisture Content (% Wet Basis)	Bulk Density (kg/m^3^)	Tapped Density (kg/m^3^)	True Density (kg/m^3^)	Abrasive Hardness Index	Mean Kernel Hardness Index	Mean Kernel Diameter (mm)	Mean Kernel Weight (mg)
Untempered Grain	13.46 (0.08)	792.26 (0.06) a	862.13 (0.00) a	1372.53 (2.56) a	22.65 (1.72) a	73.18 (0.78) a	2.95 (0.01) d	24.29 (0.27) b
RW—16%—24 h	16.10 (0.12)	787.65 (0.73) b	847.61 (0.04) b	1366.07 (0.47) b	22.25 (1.17) a	71.23 (1.47) abcd	3.07 (0.01) cd	25.44 (0.11) bac
RW—18%—24 h	18.09 (0.08)	782.15 (1.23) c	832.55 (0.04) c	1351.17 (1.21) e	21.43 (0.00) a	71.41 (0.24) abc	3.11 (0.02) c	24.86 (0.41) bac
HW—16%—12 h	15.73 (0.07)	786.41 (0.67) b	847.55 (0.06) b	1364.80 (2.18) cb	23.60 (0.00) a	71.03 (0.11) abcd	3.06 (0.05) cd	24.34 (0.59) bac
HW—16%—18 h	15.33 (0.08)	788.12 (1.19) b	847.58 (0.06) b	1364.40 (2.27) cb	23.60 (0.00) a	71.75 (0.57) ab	3.06 (0.02) cd	24.47 (0.55) bac
HW—16%—24 h	15.54 (0.06)	783.10 (0.63) c	847.56 (0.05) b	1360.93 (1.47) c	23.34 (0.37) a	71.97 (0.00) ab	3.06 (0.05) cd	24.56 (0.75) bac
HW—18%—12 h	17.74 (0.53)	771.90 (1.07) d	833.46 (0.09) c	1355.87 (2.32) d	22.13 (0.99) a	69.47 (1.30) bcd	3.25 (0.04) b	25.89 (0.16) ba
HW—18%—18 h	17.79 (0.56)	768.00 (0.34) f	826.61 (0.01) d	1354.90 (2.09) ed	21.43 (0.00) a	68.82 (0.56) bcd	3.24 (0.05) b	25.13 (0.02) bac
HW—18%—24 h	17.78 (0.02)	769.01 (0.89) ef	833.44 (0.08) c	1354.53 (0.74) ed	23.73 (0.19) a	68.27 (0.00) cd	3.23 (0.00) b	25.23 (0.19) bac
S—5 s	16.89 (0.09)	770.64 (0.32) ed	833.53 (0.04) c	1351.07 (2.19) e	21.43 (0.00) a	68.01 (1.32) d	3.13 (0.01) cb	24.77 (0.29) bac
S—10 s	21.19 (0.08)	763.30 (0.99) g	793.81 (0.01) e	1337.47 (1.42) f	21.32 (0.16) a	62.03 (1.47) e	3.09 (0.04) c	23.85 (0.70) ba
S—15 s	22.26 (0.13)	739.08 (0.81) h	781.40 (0.06) f	1325.13 (1.37) g	22.37 (1.01) a	56.43 (0.48) f	3.45 (0.06) a	26.10 (0.61) a

RW = room temp water, HW = hot water, S = steam. Values in the parentheses are standard deviations. The same lower-case letter in the same column indicates no significant difference (*p* ≤ 0.05).

**Table 3:** Angle of repose and coefficients of friction of untempered and tempered white sorghum.

Treatment	Angle of Repose (°)	Coefficient of Static Friction	Coefficient of Rolling Friction
Untempered grain	17.54 (0.36) d	0.17 (0.06) c	0.24 (0.01) d
RW—16%—24 h	19.45 (0.23) dc	0.23 (0.04) cb	0.25 (0.01) d
RW—18%—24 h	18.77 (0.34) d	0.23 (0.06) cb	0.27 (0.00) c
HW—16%—12 h	19.03 (0.31) d	0.27 (0.00) b	0.25 (0.01) d
HW—16%—18 h	19.44 (0.83) dc	0.25 (0.03) b	0.26 (0.01) d
HW—16%—24 h	21.02 (1.03) bc	0.28 (0.03) b	0.25 (0.01) d
HW—18%—12 h	21.30 (1.01) bac	0.28 (0.03) b	0.27 (0.01) cb
HW—18%—18 h	21.50 (0.87) bac	0.27 (0.00) b	0.28 (0.01) cb
HW—18%—24 h	21.57 (0.94) ba	0.28 (0.00) b	0.29 (0.01) b
S—5 s	21.49 (1.68) bac	0.29 (0.04) b	0.29 (0.01) b
S—10 s	21.59 (1.20) ba	0.29 (0.04) b	0.29 (0.01) b
S—15 s	23.20 (0.95) a	0.29 (0.04) b	0.32 (0.01) a

RW = room temperature water, HW = hot water, S = steam. Values in the parentheses are standard deviations. The same lower-case letter in the same column indicate no significant difference (*p* ≤ 0.05).

**Table 4:** Milling outcomes of white sorghum from the developed flowsheet.

Treatment	Flour Yield (g/100 g)	Bran (g/100 g)	Fine Bran (g/100 g)	Shorts (g/100 g)	Red Dog (g/100 g)
RW—16%—24 h	59.87 (0.43) abc	3.83 (0.18) fg	0.29 (0.05) f	18.64 (0.69) ab	12.59 (0.00) a
RW—18%—24 h	62.08 (0.04) ab	6.55 (0.21) c	0.53 (0.01) de	14.83 (0.13) e	11.31 (0.55) bc
HW—16%—12 h	61.01 (0.89) ab	4.10 (0.17) f	0.30 (0.07) f	17.65 (0.12) abc	12.30 (0.40) ab
HW—16%—18 h	62.96 (0.97) a	3.67 (0.13) g	0.28 (0.03) f	17.46 (0.39) bc	12.59 (0.55) a
HW—16%—24 h	60.71 (0.08) abc	3.58 (0.14) g	0.59 (0.02) cde	18.86 (0.85) a	11.88 (0.32) ab
HW—18%—12 h	61.25 (1.41) ab	5.43 (0.07) d	0.70 (0.01) bc	16.67 (0.04) cd	11.91 (0.01) ab
HW—18%—18 h	56.44 (1.87) d	6.73 (0.02) c	0.62 (0.01) bcd	16.94 (0.13) c	11.54 (0.29) abc
HW—18%—24 h	59.99 (0.16) abc	5.68 (0.14) d	0.74 (0.02) b	16.55 (0.53) cd	11.58 (0.17) abc
S—5 s	61.59 (1.37) ab	4.97 (0.04) e	0.47 (0.03) e	18.25 (0.21) ab	12.59 (0.10) a
S—10 s	59.50 (0.63) bc	8.13 (0.13) b	0.49 (0.08) de	15.59 (0.31) ed	10.85 (0.25) cd
S—15 s	57.91 (0.54) cd	10.12 (0.02) a	0.89 (0.06) a	15.51 (0.15) ed	10.05 (0.21) d

RW = room temperature water, HW = hot water, S = steam. Values in the parentheses are standard deviations. The same lower-case letter in the same column indicates no significant difference (*p* ≤ 0.05).

**Table 5:** Proximate composition and damaged starch content of milled white sorghum flour.

Treatment	Total Starch (g/100 g)	Protein (g/100 g)	Ash (g/100 g)	Crude Fat (g/100 g)	Crude Fiber (g/100 g)	Damaged Starch (g/100 g)
RW—16%—24 h	82.91 (2.42) bc	8.66 (0.06) abc	0.95 (0.02) bc	0.82 (0.05) d	0.56 (0.01) a	5.49 (0.21) de
RW—18%—24 h	81.38 (1.53) c	8.55 (0.17) abc	0.63 (0.04) e	0.42 (0.05) e	0.32 (0.04) d	5.25 (0.06) e
HW—16%—12 h	81.18 (1.79) c	8.72 (0.12) ab	0.92 (0.04) c	1.06 (0.01) c	0.44 (0.05) bc	6.65 (0.31) b
HW—16%—18 h	84.81 (1.75) abc	8.78 (0.11) a	1.02 (0.03) ab	1.98 (0.01) a	0.55 (0.01) a	5.80 (0.21) cd
HW—16%—24 h	84.76 (1.76) abc	8.49 (0.20) abc	0.93 (0.04) bc	1.70 (0.04) b	0.50 (0.03) ab	7.38 (0.22) a
HW—18%—12 h	85.13 (0.46) abc	8.35 (0.09) c	0.87 (0.04) c	1.83 (0.06) b	0.32 (0.01) d	6.71 (0.12) b
HW—18%—18 h	85.25 (1.50) abc	8.39 (0.14) bc	1.04 (0.04) a	1.78 (0.07) b	0.55 (0.02) a	7.10 (0.07) ab
HW—18%—24 h	87.23 (1.93) ab	8.36 (0.04) c	0.94 (0.03) bc	1.10 (0.06) c	0.59 (0.04) a	6.16 (0.07) c
S—5 s	86.69 (2.37) ab	8.45 (0.10) abc	0.75 (0.01) d	0.36 (0.04) ef	0.38 (0.02) cd	6.02 (0.09) c
S—10 s	86.99 (1.76) ab	7.83 (0.16) d	0.70 (0.01) de	0.23 (0.05) f	0.33 (0.05) d	7.08 (0.01) ab
S—15 s	88.20 (1.18) a	7.61 (0.09) d	0.66 (0.02) e	0.20 (0.04) f	0.39 (0.04) cd	7.35 (0.08) a

RW = room temperature water, HW = hot water, S = steam. Values in the parentheses are standard deviations. The same lower-case letter in the same column indicates no significant difference (*p* ≤ 0.05).

**Table 6:** Proximate composition of untempered white sorghum kernels.

Composition	Untempered White Sorghum Kernel (*w*/*w*%)
Total Starch	59.64 (0.79)
Protein	11.34 (0.12)
Ash	1.61 (0.02)
Crude Fiber	2.30 (0.00)
Crude Fat	1.61 (0.02)

Values in the parentheses are standard deviations.

**Table 7:** Moisture content, density (bulk, tapped and true densities), and color values of milled white sorghum flour.

Treatment	Moisture Content (% w.b.)	Bulk Density (kg/m^3^)	Tapped Density (kg/m^3^)	True Density (kg/m^3^)	L (Lightness)	a (Green to Red)	b (Blue to Yellow)
RW—16%—24 h	13.08 (0.49) d	451.90 (3.93) d	555.05 (5.71) c	1456.10 (0.87) d	84.64 (0.29) e	0.32 (0.03) bc	10.15 (0.08) d
RW—18%—24 h	15.14 (0.52) bc	467.15 (1.79) c	567.30 (1.97) b	1464.72 (1.30) bc	86.08 (0.38) cd	0.25 (0.03) d	10.11 (0.08) d
HW—16%—12 h	13.22 (0.10) d	446.07 (2.03) e	553.79 (3.68) c	1456.20 (0.57) d	84.67 (0.00) e	0.29 (0.01) cd	10.70 (0.03) c
HW—16%—18 h	13.77 (0.03) d	443.67 (1.95) e	556.09 (3.93) c	1454.83 (2.35) d	84.78 (0.29) e	0.38 (0.01) b	11.17 (0.10) b
HW—16%—24 h	13.78 (0.07) d	455.00 (2.39) d	552.78 (4.03) c	1457.55 (0.64) d	85.69 (0.17) d	0.33 (0.03) bc	11.69 (0.27) a
HW—18%—12 h	14.68 (0.14) c	463.61 (1.73) c	565.76 (0.55) b	1465.95 (2.47) bc	86.55 (0.28) bc	0.35 (0.02) b	10.93 (0.21) bc
HW—18%—18 h	15.20 (0.14) bc	466.34 (0.66) c	567.11 (0.38) b	1462.20 (1.82) c	84.67 (0.21) e	0.54 (0.04) a	11.06 (0.07) b
HW—18%—24 h	15.49 (0.03) b	469.03 (0.44) c	568.32 (0.09) b	1463.80 (1.14) bc	86.19 (0.07) bcd	0.37 (0.04) b	10.89 (0.02) bc
S—5 s	13.78 (0.23) d	456.88 (1.64) d	567.86 (4.05) b	1467.95 (2.90) b	86.94 (0.26) ab	0.32 (0.03) bc	10.38 (0.08) d
S—10 s	15.37 (0.42) bc	478.62 (1.63) b	577.06 (1.79) a	1475.50 (0.53) a	87.68 (0.21) a	0.15 (0.02) e	9.40 (0.11) e
S—15 s	17.72 (0.19) a	484.06 (0.72) a	581.40 (1.95) a	1478.83 (1.27) a	87.48 (0.20) a	0.06 (0.01) f	9.08 (0.03) f

RW = room temperature water, HW = hot water, S = steam. Values in the parentheses are standard deviations. The same lower-case letter in the same column indicates no significant difference (*p* ≤ 0.05).

**Table 8:** Properties of baked white sorghum bread.

Tempering Condition	Bake Loss (%)	Volume Index (mm)	Slice Brightness	Number of Crumb Cells	Cell Wall Thickness (mm)	Crumb Cell Diameter (mm)	Firmness (g)	Resilience (%)
RT—16%—24 h	9.51 (0.73) abc	167.00 (2.83) cd	121.65 (0.21) bcd	1535.00 (63.64) e	0.37 (0.03) a	1.15 (0.05) a	1234.81 (9.81) ab	47.63 (0.60) abc
RT—18%—24 h	10.59 (0.57) ab	176.50 (2.12) ab	124.73 (3.72) abc	2051.00 (93.34) a	0.35 (0.02) a	0.95 (0.08) b	1398.79 (23.13) a	54.25 (0.20) a
HW—16%—12 h	9.02 (0.66) bc	163.00 (4.24) cde	124.73 (3.72) abc	1778.00 (60.81) bcd	0.36 (0.02) a	1.02 (0.07) ab	1241.46 (15.44) ab	51.07 (1.17) abc
HW—16%—18 h	10.65 (0.40) ab	165.00 (1.41) cde	114.43 (2.21) de	1915.33 (32.62) ab	0.34 (0.00) a	1.00 (0.03) ab	1293.46 (35.43) ab	50.22 (0.36) abc
HW—16%—24 h	10.41 (0.29) ab	160.00 (2.83) de	114.50 (4.95) de	1647.50 (41.72) cde	0.35 (0.02) a	1.04 (0.08) ab	1283.26 (91.97) ab	50.58 (1.24) abc
HW—18%—12 h	10.72 (0.61) a	165.50 (0.71) cde	114.63 (2.22) de	1994.00 (63.64) a	0.35 (0.01) a	1.03 (0.06) ab	1234.53 (98.34) ab	42.42 (0.55) c
HW—18%—18 h	10.22 (0.50) ab	156.50 (3.54) e	109.50 (1.41) e	1863.50 (84.15) abc	0.34 (0.01) a	0.99 (0.02) ab	1093.43 (12.42) bc	46.71 (0.31) abc
HW—18%—24 h	8.31 (0.67) c	170.33 (2.89) bc	117.63 (1.76) cde	1739.00 (73.54) bcde	0.36 (0.01) a	1.10 (0.06) ab	1229.65 (49.86) ab	52.04 (2.04) ab
S—5 s	9.12 (0.54) bc	178.50 (2.12) ab	128.45 (0.64) ab	1671.50 (21.92) cde	0.37 (0.01) a	1.07 (0.04) ab	1089.31 (18.53) bc	46.47 (5.00) abc
S—10 s	9.80 (0.12) abc	178.67 (0.58) ab	127.27 (2.78) ab	1770.00 (48.08) bcd	0.37 (0.02) a	1.05 (0.03) ab	1026.78 (12.42) bc	43.71 (3.28) bc
S—15 s	9.96 (0.50) ab	179.50 (3.54) a	131.60 (2.40) a	1612.50 (62.93) de	0.38 (0.00) a	1.12 (0.02) ab	1003.12 (107.48) c	50.31 (0.74) abc

RT = room temperature water, HW = hot water, S = steam. Values in the parentheses are standard deviations. The same lower-case letter in the same column indicates no significant difference (*p* ≤ 0.05).

## Data Availability

The data presented in this study are available on request from the Corresponding author.
